# Protein-Energy Malnutrition Increases Mortality in Patients Hospitalized With Bacterial Pneumonia: A Retrospective Nationwide Database Analysis

**DOI:** 10.7759/cureus.12645

**Published:** 2021-01-12

**Authors:** Sriram Gonakoti, Iyobosa F Osifo

**Affiliations:** 1 Internal Medicine, John H. Stroger, Jr. Hospital of Cook County, Chicago, USA; 2 Medicine, Aminu Kano Teaching Hospital, Kano, NGA

**Keywords:** community acquired pneumonia, protein energy malnutrition (pem), respiratory bacteria, morbidity and mortality

## Abstract

Background

Malnutrition is a less commonly recognized risk factor for various infections. It encompasses both undernutrition or protein-energy malnutrition (PEM) and overnutrition, including obesity. This study aimed to evaluate whether PEM impacts bacterial pneumonia (BP) and, if so, to quantify the degree of impact on inpatient outcomes.

Methods

This was a retrospective cohort study involving adult hospitalizations for BP using the nationwide inpatient database. Outcomes included comparing inpatient mortality, total hospital charges, length of hospital stay, as well as complications from bacterial pneumonia.

Results

The in-hospital mortality for adults with BP was 2.62%. Patients with PEM had a higher adjusted odds ratio (aOR) of inpatient mortality (adjusted odds ratio (aOR): 2.31, 95% confidence interval (CI): 2.14 - 2.48, p<0.001) as compared to non-PEM patients. PEM was also associated with higher odds of sepsis (aOR: 2.24, 95% CI: 2.04 - 2.46, p<0.001), septic shock (aOR: 3.29, 95% CI: 2.82 - 3.85, p<0.001), requiring mechanical ventilation (aOR: 2.51, 95% CI: 2.31 - 2.71, p<0.001), requiring vasopressors (aOR: 2.90, 95% CI: 2.20 - 3.83, p<0.001), acute respiratory distress syndrome (ARDS) (aOR: 1.63, 95% CI: 1.33 - 2.00, p<0.001), acute kidney failure (AKI) (aOR: 1.24, 95% CI: 1.18 - 1.29, p<0.001), deep vein thrombosis (DVT) (aOR: 1.80, 95% CI: 1.62 - 2.00, p<0.001), and pulmonary embolism (PE) (aOR: 1.25, 95% CI: 1.08 - 1.45, p=0.003).

Conclusion

The study concluded that PEM was an independent mortality predictor for those with BP, with an increased risk of systemic complications, as well as increased healthcare utilization costs.

## Introduction

Community-acquired pneumonia (CAP) is defined as an acute infection of the lung parenchyma acquired outside of the hospital. In the United States, pneumonia accounts for over 1.2 million emergency room visits annually [1]. Risk factors for pneumonia include older age, diabetes mellitus, chronic lung diseases (e.g., asthma, chronic obstructive pulmonary disease, bronchiectasis), heart failure, smoking, and alcohol use. CAP may have bacterial or viral causes [2].

Malnutrition is a less commonly recognized risk factor for various infections. It encompasses both undernutrition or protein-energy malnutrition (PEM) and overnutrition, including obesity. Malnutrition has been reported to increase the risk of infection and impacts the hospital outcomes of various disease conditions [2-9]. The World Health Organization (WHO) estimates that 462 million adults have PEM [10]. Diagnostic criteria have been set forth by the Academy of Nutrition and Dietetics (Academy) and the American Society for Parenteral and Enteral Nutrition (ASPEN), which defines PEM as two or more of the following: insufficient energy intake, weight loss, loss of muscle mass, loss of subcutaneous fat, a localized or generalized fluid accumulation that may mask weight loss, and diminished functional status as measured by handgrip strength [11]. PEM in the adult population has been shown to increase health care utilization, with longer hospital stays and poor functional outcomes [12].

There is a paucity of large population-based studies on the effect of PEM on bacterial pneumonia. Hence, this study aimed to evaluate whether PEM impacts bacterial pneumonia (BP) and, if so, to quantify the degree of impact on inpatient outcomes.

## Materials and methods

Design and data source

This was a retrospective cohort study involving adult hospitalizations principally for bacterial pneumonia (BP) in the US in 2016 and 2017. Data were obtained from the Nationwide Inpatient Sample (NIS) database. The NIS is a database of hospital inpatient stays derived from billing data submitted by hospitals to statewide data organizations across the US, covering more than 97% of the U.S. population [13]. It approximates a 20% stratified sample of discharges from U.S. community hospitals, excluding rehabilitation and long-term acute-care hospitals. This dataset is weighted to obtain national estimates [14]. The database was coded using the International Classification of Diseases, Tenth Revision, Clinical Modification/Procedure Coding System (ICD-10-CM/PCS). The NIS has primary and secondary diagnostic codes corresponding to the principal reason for admission and all other discharge diagnoses, respectively.

Study population

We queried the NIS 2016 and 2017 databases for patients 18 years and above who had a principal discharge diagnosis of PB. From the literature review, prior studies utilized ICD-10 codes for BP, including J13, J14, and J15 [15]. This cohort was divided based on the presence of a secondary discharge diagnosis of PEM.

Outcome measures

Inpatient mortality between non-PEM and PEM with BP groups was assessed as the primary outcome. Secondary outcomes assessed included odds of sepsis, septic shock, acute hypoxic respiratory failure (ARF), acute respiratory distress syndrome (ARDS), non-ST segment elevation myocardial infarction (NSTEMI), acute kidney failure (AKI), deep vein thrombosis (DVT), pulmonary embolism (PE), cerebrovascular accident (CVA), need for mechanical ventilation, vasopressors, as well as mean length of hospital stay (LOS) and mean total hospital charges (THC).

Statistical analysis

We analyzed the data using Stata® Version 16 software (StataCorp, Texas). The proportions of comorbid diseases and baseline characteristics were compared using chi-squared tests. Multivariate regression analysis was done to adjust for possible confounders while calculating the primary and secondary outcomes. These were obtained from prior related studies reviewed and included variables from the validated pneumonia severity index [16]. A univariate screen was done to further confirm these factors affected outcomes with variables having a p-value less than 0.2 included in the multivariate analysis [17-20]. The threshold for statistical significance was 0.05.

Ethical considerations

The NIS database lacks patient identifiers and contains only retrospective data. Hence, this study was exempt from Institutional Review Board approval.

## Results

Patient characteristics

Table 1 contains patient and hospital characteristics of hospitalizations involving adults with BP. A total of 1,236,109 hospitalizations were studied. Compared to non-PEM patients, those with PEM had an older mean age (71.8 vs 68.4 years, p<0.001) with a lower proportion of women (51.0 vs 53.3%, p<0.001). Patients with PEM had higher proportions of CKD (18.8 vs 16.9%, p<0.001), chronic obstructive pulmonary disease (COPD) (37.5 vs 31.5%, p<0.001), and anemia (46.8 vs 27.1%, p<0.001). However, they had lower proportions of hypertension (36.1 vs 42.6%, p<0.001), diabetes (24.0 vs 31.6%, p<0.001), and dyslipidemia (31.6 vs 37.4%, p<0.001).

**Table 1:** Patient and hospital characteristics of hospitalizations for bacterial pneumonia by PEM #: for 2017. CHF: Congestive heart failure, CKD: Chronic kidney disease, COPD: Chronic obstructive pulmonary disease, CVA: Cerebrovascular accident, IHD: Ischemic heart disease, PEM: Protein-energy malnutrition

Variable	PEM %	Without PEM %	p-value
N= 1,236,109	n= 86,585 (7.0)	n= 1,149,524 (93.0)	
Patient characteristics			
Age, mean years	71.8	68.4	<0.001
Women	51.0	53.3	<0.001
Racial distribution			<0.001
White	71.0	71.8	
Black	12.2	11.8	
Hispanic	7.2	7.9	
Others	9.6	8.5	
Insurance type			<0.001
Medicaid	75.7	69.2	
Medicare	10.4	10.9	
Private	12.3	16.9	
Uninsured	1.6	3.0	
Charlson Comorbidity Index score			<0.001
0	10.3	17.3	
1	22.0	25.7	
2	19.6	19.5	
≥3	48.1	37.5	
Median annual income in patient’s zip code, US$^#^			0.013
1-43,999	33.4	33.1	
44,000-55,999	26.6	28.0	
56,000-73,999	22.7	22.1	
≥74,000	17.2	16.8	
Co-morbidities			
Hypertension	36.1	42.6	<0.001
Diabetes	24.0	31.6	<0.001
Smoking history	43.2	42.0	0.004
CHF	25.9	25.0	0.010
CKD	18.8	16.9	<0.001
Dyslipidemia	31.6	37.4	<0.001
Chronic IHD	24.4	25.4	0.004
Prior CVA	3.7	2.6	<0.001
COPD	37.5	31.5	<0.001
Oxygen dependent	10.6	8.6	<0.001
History of malignancy	24.8	12.0	<0.001
Anemia	46.8	27.1	<0.001
Hospital characteristics			
Hospital region			<0.001
Northeast	15.5	17.9	
Midwest	25.1	24.3	
South	40.1	41.9	
West	19.3	15.9	
Hospital bed size			<0.001
Small	22.1	27.1	
Medium	30.4	30.3	
Large	47.5	42.6	
Urban location	85.7	80.1	<0.001
Teaching hospital	57.1	50.9	<0.001

Outcomes in patients with PEM

The in-hospital mortality for adults with BP was 2.62%. Patients with PEM had a higher adjusted odds ratio (aOR) of inpatient mortality (aOR: 2.31, 95% confidence interval (CI): 2.14 - 2.48, p<0.001) as compared to non-PEM patients. PEM was also associated with higher odds of sepsis (aOR: 2.24, 95% CI: 2.04 - 2.46, p<0.001), septic shock (aOR: 3.29, 95% CI: 2.82 - 3.85, p<0.001), requiring mechanical ventilation (aOR: 2.51, 95% CI: 2.31 - 2.71, p<0.001), requiring vasopressors (aOR: 2.90, 95% CI: 2.20 - 3.83, p<0.001), ARDS (aOR: 1.63, 95% CI: 1.33 - 2.00, p<0.001), AKI (aOR: 1.24, 95% CI: 1.18 - 1.29, p<0.001), DVT (aOR: 1.80, 95% CI: 1.62 - 2.00, p<0.001), and PE (aOR: 1.25, 95% CI: 1.08 - 1.45, p=0.003). Patients with PEM also had a significantly longer LOS, as well as higher THC, as compared to non-PEM patients (Table 2).

**Table 2:** Clinical outcomes in patients with bacterial pneumonia and PEM *; statistically significant, #; Adjusted mean difference, aOR: Adjusted odds ratio, CI: Confidence interval, ARDS: Acute respiratory distress syndrome, NSTEMI; Non-ST segment elevation myocardial infarction, PEM: Protein-energy malnutrition

Outcome	PEM %	Without PEM %	aOR (95% CI)	p-value*
N= 1,236,109	n= 86585 (7.0)	n= 1,149524 (93.0)		
Primary outcome				
In-hospital mortality	6.8	2.3	2.31 (2.14 – 2.48)	<0.001*
Secondary outcomes				
Length of stay, mean	7.4	4.6	2.4^ # ^(2.2 – 2.5)	<0.001*
Total hospital charges, mean US$	63500	36100	21800^# ^(20100 - 23400)	<0.001*
Sepsis	4.2	1.6	2.24 (2.04 – 2.46)	<0.001*
Septic shock	1.5	0.3	3.29 (2.82 – 3.85)	<0.001*
NSTEMI	1.6	1.2	1.10 (0.96 – 1.26)	0.158
Mechanically ventilated	6.7	2.4	2.51 (2.31 – 2.71)	<0.001*
Used pressors	0.5	0.1	2.90 (2.20– 3.83)	<0.001*
Acute kidney failure	20.4	14.9	1.24 (1.18 – 1.29)	<0.001*
Acute respiratory failure	20.6	15.9	1.29 (1.23 – 1.34)	<0.001*
ARDS	0.7	0.4	1.63 (1.33 – 2.00)	<0.001*
Deep vein thrombosis	2.8	1.1	1.80 (1.62 – 2.00)	<0.001*
Pulmonary embolism	1.2	0.7	1.25 (1.08 – 1.45)	0.003*
Cerebrovascular accident	0.6	0.3	1.53 (1.22 – 1.91)	<0.001*

## Discussion

Risk factors for PEM in adults include age, frailty in institutionalized persons, excessive polypharmacy, general health decline, including physical function, Parkinson disease, constipation, poor or moderate self-reported health status, cognitive decline, dementia, eating dependencies, poor appetite, basal oral dysphagia, signs of impaired efficacy of swallowing, and institutionalization [21]. Synergism exists between malnutrition and infection, wherein malnutrition increases the risk of infection, and infection can lead to malnutrition. This leads to the formation of a vicious cycle. Our study reveals an increased incidence of chronic medical conditions such as chronic kidney disease, chronic obstructive pulmonary disease, and anemia in patients with PEM.

Our study demonstrated PEM as an independent mortality predictor for those with bacterial pneumonia. The estimated mortality in patients with PEM that are admitted with bacterial pneumonia is over two times higher when compared to those without PEM. Patients with PEM are at a higher risk of complications from bacterial pneumonia, including sepsis, septic shock, acute respiratory distress syndrome, acute kidney injury, deep vein thrombosis, and pulmonary embolism.

Various factors could explain the findings of this study. Malnutrition is the most common cause of secondary immunodeficiency, affecting up to 50% of at-risk populations [22]. Malnutrition affects both cell-mediated and humoral immunity. It leads to a reduction in the number of antigen-presenting-cells, such as B-lymphocytes, Kupffer cells, macrophages, and dendritic cells. The complement system is also affected, limiting the phagocytic function. Severe protein-energy malnutrition in neonates and infants was associated with atrophy of bone marrow and the thymus, resulting in low B and T lymphocytes. Also, there is reduced activation of T-lymphocytes. Changes in the microanatomy of barrier epithelium also lead to an increased risk of infection [23].

Our study was based on the NIS administrative database, where the diagnosis was based on ICD-10 codes and not microbiologic confirmation, which may affect the data [24-26]. The number of patients with protein-energy malnutrition may be underrepresented owing to under coding in an acute setting. A patient may have multiple admissions reflected in the NIS. NIS studies cannot determine causation, only potential association. These are important limitations of this study.

## Conclusions

PEM is an independent mortality predictor for those with bacterial pneumonia, with an increased risk of systemic complications as well. There is a need for a multidisciplinary approach against malnutrition, which can mitigate the risk, leading to lower mortality, morbidity, and health care expenditure.
